# Fit for purpose - developing a software platform to support the modern challenges of data linkage in Western Australia

**DOI:** 10.23889/ijpds.v3i3.435

**Published:** 2018-11-12

**Authors:** Tom Walter Eitelhuber, James Thackray, Steve Hodges, Janine Alan

**Affiliations:** 1 The Western Australian Department of Health, Data Linkage Branch

## Abstract

The Western Australia Data Linkage System (WADLS) is maintained and operated by the WA Data Linkage Branch (DLB) at the Western Australian Department of Health. DLB has pioneered a number of data linkage innovations, including the facilitation of genealogical research via the Family Connections system and streamlined data delivery via the Custodian Administered Research Extract Server. DLB’s latest innovation is a new data linkage system called “DLS3”, which improves DLB’s capability and capacity to handle the increasing volume and complexity of its routine operations. DLS3 was built entirely in-house and customised to meet the specific challenges that DLB has encountered throughout over twenty years of experience with a wide variety of linkages. This article describes the development and rollout of DLS3, including its design, architecture, benefits and limitations.

## Data linkage in Western Australia

Western Australia (WA) covers approximately a third (2.5M square kilometres) of Australia’s total landmass and 10 per cent (2.6M people) of its population [1]. WA’s health system comprises both public and private health services, including public-private partnerships. The WA Department of Health is responsible for the overall management, performance, and strategic direction of the WA public health system to ensure the delivery of high-quality, safe, timely and accessible health services [2]. The WA Data Linkage Branch (DLB) is part of the WA Department of Health, and it develops and maintains the WA Data Linkage System (WADLS).

Data linkage is the practice of connecting pieces of information thought to belong to the same person, place, family or event [3]. Data linkage can facilitate research, planning, policy development and evaluation of services, and it has delivered a variety of benefits in Australia and internationally by enabling complex, multi-jurisdictional analysis for a range of purposes [4].

Initially established in 1995[5], the WADLS is the longest operating Australian linkage system and it is underpinned by strong privacy and security principles [6,7]. Initially comprising just a few data collections, the WADLS has grown considerably over the past two decades. It now comprises over 100 million records spanning approximately 50 linked datasets. New datasets are imported and linked on a regular basis, including hospitalisations, emergency attendances, births, deaths, electoral enrolments and a variety of other health and non-health information. Schedules for linkage updates vary (weekly, monthly, quarterly, annual or irregular), depending on resourcing constraints and the availability of, and demand for, a particular dataset. Many datasets are linked at a higher frequency to ensure up-to-date information is available, and some datasets include records dating as early as 1945[8]. The WADLS structure consists of two sets of database tables – a demographic database where the identifying information required for linkage is loaded and stored; as well as the separate links database where the linkage keys are stored, managed and extracted as required to service a variety of requests.

DLB releases tailored, linked data extracts directly to Data Applicants, with the personal identifiers removed, for requests that are granted the applicable ethical, research and data governance approvals [9]. The linkage keys are encrypted, with a different encryption key used for each request. Occasionally, approval may not be granted for the release of data directly to the applicant, for example if an appropriate level of security is not assured, or if direct release is otherwise prohibited (e.g. by legislation). In these instances, access to linked data may be enabled via alternate models, potentially involving third party secure environments, other linkage agencies or Integrating Authorities [10]. The WADLS, including the newly developed linkage system, can service any of these models and is agnostic to the release method employed.

WA lacks a population-wide personal unique identifier (e.g. social security number) that can be used for linkage. To overcome this challenge, DLB uses probabilistic linkage processes, where groups of records are compared using complex field matching algorithms. These algorithms compare a user-defined list of common fields, such as given name, surname, date of birth and address (although the exact list of fields to be considered varies depending on the contents and context of the datasets involved) and provide a score indicating their similarity. Most algorithms also incorporate user-defined parameters and frequency review to refine the score. These field-wise scores are combined to provide an overall match score for each pair of records under consideration, indicating the likelihood that the two records belong to the same individual [6].

Each dataset linked by DLB has its own formatting, quality, completeness and other idiosyncrasies, which depend upon the context, standards and capabilities at the point of collection. Linkage is often best achieved via a customised approach to harness the strengths and minimise the quality issues associated with each dataset. Over time, these customised processes are maintained and enhanced to ensure the stability and suitability of ongoing linkage updates as the source dataset evolves.

## The impetus for change

Historically, DLB’s legacy linkage system used proprietary file-based linkage software (referred to as “FLS” hereafter) to perform the linkage process. The FLS was identified in isolation because its features were key to the legacy system and its limitations were difficult or impossible to avoid. The legacy system (which, along with DLB’s earliest prototype system, was never formally named) grew organically to address the requirements of DLB.

Linkage using this FLS involved the following steps, each customised by the user:

1. Two datasets were chosen for comparison
2. Pairs of records meeting user-defined criteria were sub-setted (known as *blocking*)
3. These record pairs were evaluated using user-defined matching algorithms (*matching*)
4. Based on the outcomes of the matching algorithms, weights were assigned representing the likelihood that the two records belonged to the same person (*weights assignment*)
5. Records were categorised as ‘match’, ‘non-match’ or ‘potential match’ based on user-defined weight thresholds (*threshold setting*)

Matches and potential matches were submitted for quality review protocols [11], including a phase of manual scrutiny for potential matches (clerical review), wherein a DLB Linkage Officer visually reviewed the potential pairings via a user interface and decided, using any available contextual information, whether to match the records. Following this phase, links were loaded into the WADLS. This process was then repeated for a new subset of fields and/or pair of datasets.

The FLS used by DLB was well-suited to the task of linking datasets of modest size and complexity during DLB’s formative years. However, as the WADLS grew, a number of key limitations and issues with the FLS’s capabilities became evident (Table 1).

**Table 1: Limitations of the File-based Linkage Software (FLS) previously used by the Data Linkage Branch:** 

File-based Linkage Software (FLS) Limitation	
Discreteness	FLS did not effectively retain or share information about matching decisions, which led to repetition
Iterative process	FLS required linkages to run in a set order, with the clerical review of one phase completed prior to the next phase starting. This approach required substantial manual input by the Linkage Officer. Sometimes, this led to a less efficient strategy being used to determine a link, when a better option may have become apparent during a subsequent phase of the linkage process
Pairwise matching	FLS could not concurrently consider more than two records for potential matching, which prevented the Linkage Officer from leveraging the breadth and depth of the WADLS without additional bespoke tools (if feasible and available) and manual intervention
Change control	FLS software lacks integrated change control, which means adjustments to linkage protocols over time are not captured easily
Proprietary concerns	FLS was subsumed into a new product, which introduced issues associated with cost, compatibility, support and maintenance

Furthermore, the FLS historically used by DLB was only designed to compare records and generate match weights. It did not include any functionality to support a variety of other important linkage functions, including: data cleaning and standardisation; importation, storage and extraction of data; loading, storage and extraction of links; clerical review processes; quality checking processes; and maintaining system metadata such as the history of decisions. These functions were supported via DLB’s customised software modules that were not fully integrated with the FLS. While these bespoke DLB software solutions improved the flexibility, power and efficiency of the linkage framework, dependency on the FLS was limiting DLB’s progress. This recognition provided impetus to develop a state-of-the-art system, tailored to meet DLB’s unique requirements.

## Objectives and considerations in the design stage

DLS3, short for ‘Data Linkage System Number 3’, follows on from the *FLS* -centric legacy system and the original prototype. DLS3 completely replaces the former FLS, many of the associated custom modules, and it will eventually replace the remaining vestiges of the legacy system. DLS3’s development was underpinned by four design pillars (Table 2).

**Table 2: Data Linkage Branch design pillars for its new data linkage system (DLS3):** 

New Data Linkage System (DLS3) Design Pillar	
Concurrency	Run all linkage strategies for a dataset concurrently
De-duplication	Remove double-handling of potential matches in the clerical review process
Streamlining	Improve the end-to-end linkage process by integrating the standardisation, matching and links management stages and removing repeated or unnecessary manual steps
Immediacy	New links would be created immediately upon being identified, rather than waiting to load a whole file of links as a batch job.

DLS3 was developed with continuous input from DLB’s operational staff. This included involvement in discussions around design and functionality, user testing and fault reports, and side-by-side evaluations with the existing linkage software (where practical) during the development and implementation stages. DLB’s subject-matter experts collectively possess almost fifty years of data linkage experience (not including staff who have since moved on) and their detailed understanding of the idiosyncrasies and challenges that DLS3 would likely encounter ensured development of a system that was fit for purpose

## System architecture and structural considerations

**Figure 1: Diagrammatical representation of DLS3 components.**
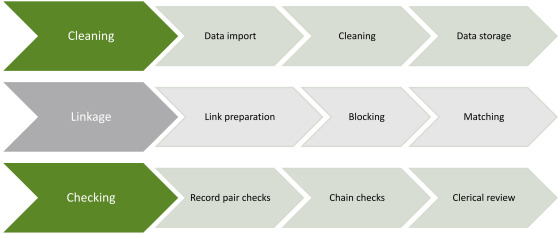


DLB designed DLS3 to comprise a number of fully integrated and function-specific components (called ‘Services’) that drive the key stages of the linkage process (Table 3 and Figure 1).

**Table 3: Structure of DLS3:** 

Data Linkage System (DLS3) Service Structure	
Loading	These three Services use dataset profiles, created by the user, that provide DLS3 with a tailored ‘recipe’ for how to standardise and store the data.
Cleaning	
Importation	
Linkage preparation	Creates subset blocks in the database for comparison, assigns initial chains to new records and processes any deleted records.
Linkage	Triggers comparison algorithms and generates match weights.
Clerical review	Prompts the user to review a de-duplicated list of all potential (i.e. non-automatic) links. Potential links are viewed as clusters of records (not individual pairs) and will only be reviewed if the cluster: (a) does not match automatically via a higher quality link identified during the linkage service; or (b) triggers a quality assurance check that overrides a higher quality link.
Extraction	Enables the user to extract linkage keys from the WADLS according to specified criteria.

DLS3 Services call upon a number of related Modules to perform functions that may span multiple Services. The two most important Modules are called ‘Checking’ and ‘Storage’, which are called by the ‘Linkage’ and ‘Clerical Review’ Services. The Checking Module runs a variety of quality assurance checks and flags suspicious potential links for further consideration. This includes threshold checks for potential matches that are assigned an ‘intermediate weight’ and require clerical review. The Storage Module stores the links in the WADLS links database, where they are managed and extracted as required.

### Integrated, scalable and powerful: the benefits of DLS3

DLB is currently operating both DLS3 and some residual parts of the legacy data linkage system, with the latter undergoing phased retirement. Prior to each retirement phase, both systems run in parallel to confirm DLS3 is working correctly and to manage any data migration risks. The FLS has been retired and DLS3 is being used exclusively for cleaning, importation and data matching. DLB is currently finalising the clerical review, links storage and extraction functions; these are the final steps before all legacy tools can be retired.

DLS3 has already yielded a number of key benefits. It creates considerable time and cost savings by running linkage strategies concurrently and delaying the clerical review stage until all potential links can be considered collectively. User input is reduced because successive stages are now integrated rather than requiring manual intervention. For example, the output of DLS3’s cleaning and importation service flows directly into its linkage preparation service.

Linkage quality is of considerable interest to data linkage centres and users of linked data [12,13]. It is typically thought of in terms of false-positives (incorrect links) and false-negatives (missed valid links), however these can be challenging to define and measure [11—13]. It is expected that the streamlining and deduplication of processes in DLS3 will ultimately realise improvements in this regard. Thorough testing has been carried out on a discrete basis to ensure no decreases in quality are evident, however a holistic review of quality across the entire WADLS cannot be undertaken until full implementation is complete and the system is in stable ongoing use.

DLB’s approach to linkage quality focuses on understanding the nature of linkage errors, implementation of robust risk-mitigation strategies and a culture of continuous improvement [11]. Following full implementation and bedding down of DLS3, DLB will consider ways to evaluate and measure the anticipated quality improvements brought about by DLS3.

Numerous matching features have been implemented in DLS3 that were previously limited or impossible in the legacy FLS environment (Table 4).

**Table 4: Matching features added or enhanced in the new data linkage system (DLS3):** 

Feature	Description & purpose	Description of legacy file-based linkage software (FLS) capability	Description of DLS3 capability
Matching functions	Used to compare different types of fields (i.e. string, numeric, date, location, etc.), including parameters for error tolerance.	New match functions cannot be added and existing functions cannot be modified.	Match functions can be designed, added and updated as required.
Matching conditions	Uses inexact comparisons and permissible field values to further restrict which records may be considered for matching.	No match conditions in FLS. Limited to sub-setting of entire input file (e.g. linking only to women).	Match conditions allow excluding some match pairs based on conditions other than exact matching (as implemented by blocking).
Data preparation functions	Clean, standardise and transform data in order to improve likelihood of matching with other datasets.	None in FLS. All data preparation must be implemented using custom add-ons developed by DLB, prior to running the FLS.	Can apply additional data cleaning or transformation at linkage stage; for example address standardisation or phonetic transforms.
Frequency calculations	Allows configuring of matching functions to give uncommon field values (e.g. surnames) more weight than common ones.	Only for 100 most frequent values.	Frequencies for all values. Can use conditional frequencies (eg. name frequencies for male vs female). Chain based rather than record based (to reduce bias in event based data).
Cardinality restrictions	Allows linkage outcomes to be restricted – one-to-one; one-to-many; many-to-one – to meet expectations of the dataset.	1:1, 1:N, N:1 matching restrictions are possible, but are limited to post-linkage checking (prior to loading links) on a record-to-record basis.	1:1, 1:N. N:1 matching restrictions are possible on a chain-to-chain basis, and can be triggered as part of a linkage strategy.
Linkage metadata	Capture current and historical information about software, systems, data and linkage keys.	No metadata recording built in. Must be done manually by users.	Metadata recording built in. Includes: Data changes Data profile changes Link changes Linkage strategy changes Linkage runs and parameters Clerical review decisions

DLB anticipates that DLS3 will continue delivering benefits as remaining modules are fully implemented and legacy tools are retired. 

### Limitations of DLS3

DLS3 has been implemented using a phased replacement of the legacy system. The original design included some enhancements to the row-level unique identifiers (for example, hospital admission event identifiers) used to identify records submitted for linkage. Due to the requirement of maintaining compatibility with DLB’s legacy FLS system (at least until it is completely replaced), it has been impossible to take advantage of enhancements in this area. DLB is confident solutions will be found to other limitations that may become apparent over time.

### Conclusion

This article has briefly described the development and rollout of its new data linkage system, DLS3, which was built entirely using the specialist skills and experience of the Data Linkage Branch in Western Australia. With *DLS3*, DLB has created an adaptable and sophisticated tool, informed by the DLB’s extensive experience and understanding of all facets of data linkage. It delivers fast, high quality, scalable and cost-effective linkages to meet the current and emerging challenges of data linkage. As the demand for wide-reaching linkage infrastructure continues to grow, it strengthens DLB’s position as a leader in data linkage innovation.

## Ethics

This publication did not require ethical approval.

## Contributions

TE and JA supervised the project. JT was lead developer on the project, supported by SH. TE drafted the original manuscript with input and critical feedback provided by JT, SH and JA.

## Abbreviations

DLB	Data Linkage Branch, Western Australian Department of Health
DLS3	Data Linkage System 3
FLS	File-based linkage software (in the context of this article, this refers to the proprietary software package used by DLB prior to the implementation of DLS3)
WADLS	Western Australian Data Linkage System
